# Patient education integrated with acupuncture for relief of cancer-related fatigue randomized controlled feasibility study

**DOI:** 10.1186/1472-6882-11-49

**Published:** 2011-06-25

**Authors:** Michael F Johnston, Ron D Hays, Saskia K Subramanian, Robert M Elashoff, Eleanor K Axe, Jie-Jia Li, Irene Kim, Roberto B Vargas, Jihey Lee, LuGe Yang, Ka-Kit Hui

**Affiliations:** 1 UCLA Center for East-West Medicine, David Geffen School of Medicine at UCLA, LA, USA; 2 Division of General Internal Medicine, David Geffen School of Medicine at UCLA, LA, USA; 3 Department of Health Services, UCLA School of Public Health, LA, USA; 4 RAND, Santa Monica, CA, USA; 5 Department of Psychiatry and Biobehavioral Sciences, David Geffen School of Medicine at UCLA, LA, USA; 6 Department of Biostatistics, School of Public Health, UCLA, LA, USA; 7 Department of Biomathematics, UCLA Graduate Division, LA, USA

## Abstract

**Background:**

Cancer-related fatigue (CRF) is a prominent clinical problem. There are calls for multi-modal interventions.

**Methods:**

We assessed the feasibility of delivering patient education integrated with acupuncture for relief of CRF in a pilot randomized controlled trial (RCT) with breast cancer survivors using usual care as control. Social cognitive and integrative medicine theories guided integration of patient education with acupuncture into a coherent treatment protocol. The intervention consisted of two parts. First, patients were taught to improve self-care by optimizing exercise routines, improving nutrition, implementing some additional evidence-based cognitive behavioral techniques such as stress management in four weekly 50-minute sessions. Second, patients received eight weekly 50-minute acupuncture sessions. The pre-specified primary outcome, CRF, was assessed with the Brief Fatigue Inventory (BFI). Secondary outcomes included three dimensions of cognitive impairment assessed with the FACT-COGv2.

**Results:**

Due to difficulties in recruitment, we tried several methods that led to the development of a tailored recruitment strategy: we enlisted oncologists into the core research team and recruited patients completing treatment from oncology waiting rooms. Compared to usual care control, the intervention was associated with a 2.38-point decline in fatigue as measured by the BFI (90% Confidence Interval from 0.586 to 5.014; p <0.10). Outcomes associated with cognitive dysfunction were not statistically significant.

**Conclusions:**

Patient education integrated with acupuncture had a very promising effect that warrants conducting a larger RCT to confirm findings. An effective recruitment strategy will be essential for the successful execution of a larger-scale trial.

**Trial registration:**

NCT00646633

## Background

Cancer-related fatigue (CRF) is an unrelenting sensation of tiredness far disproportionate to levels of physical activity. It is unmitigated by rest/sleep and is physically debilitating [1,2]. Patients with many types of cancer report CRF as their most severe symptom [3]. CRF compromises ability to complete daily routines (such as food preparation, household chores, and social activities) and impairs ability to attend/perform work [4-6]. CRF is also the most prevalent symptom [3,4]. In a systematic review, authors estimate CRF to range from 39-90+% during treatment and 19-38% after treatment [7]. CRF is multi-factorial. It appears to result from various exogenous and endogenous factors that dysregulate physiological, biochemical, and psychological systems [8,9].

Alleviating CRF is a high-priority issue, [10] with a documented need for new treatment approaches [11]. The National Comprehensive Cancer Network (NCCN) enumerates many pharmacologic and non-pharmacologic options [2]. A Cochrane [12] and associated review [11] of pharmacologic treatments for CRF finds positive results for only three: methylphindate (based on 2 small trials, one with non-significant results) and two haemopoetic growth factors: erythropoietin and darbopeitin [11,12]. The growth factors improved anemia-associated CRF, but recovery was often incomplete and was accompanied by potential side-effects [11,12]. Authors of a Cochrane review found exercise to provide some benefit, both during and after cancer treatment [13]; however authors of other reviews were less optimistic [14,15]. Addressing these discrepant interpretations, Stone and Minton [11] commented that many studies showed that exercise improved physical function without reducing fatigue. A Cochrane review [16] on psychosocial interventions during cancer treatment found limited promising evidence for several specific approaches. Though not enumerated in the NCCN recommendations, other research reports showed promising evidence from pilot studies for acupuncture [17-21] and acupressure [19,22].

Prior studies typically investigated treatment options using a single modality [23]. However, CRF often persists precisely because its complex etiology renders mono-modality approaches limited [23]. Accordingly, we drew upon elements of social cognitive theory [24] and integrative medicine theory [25-27] to create a four-session education program that involved teaching patients self-care techniques alongside an eight-session program that involved administering acupuncture. This integrated program addressed the call for new treatment options that are multimodal [28] and act in a broad-based manner [29].

This study assessed the feasibility of conducting a larger-scale randomized controlled trial (RCT) on the effectiveness of this integrated patient education and acupuncture program in relieving CRF. In doing so, we developed two specific objectives. First, to design a strategy for recruitment tailored to the institutional environment and the therapeutic intervention. This is important because successful recruitment strategies sometimes perform poorly in CAM-based research trials, [26,30-32] even though patients in the community are tremendously interested in CAM for symptom relief [33]. The second objective was to collect evidence of preliminary effects. This follows from the recommendation of a National Cancer Institute Community Clinical Oncology Program team that concluded small-scale studies are of critical importance for improving evidence-based assessments of CAM treatment regimens [34,35].

## Methods

We report study randomization procedures following the statement on Consolidated Standards of Reporting Trials (CONSORT). We generated a random allocation sequence (1:1 ratio, permuted block randomization with blocks of size four) prior to recruiting subjects using the system found at http://www.randomization.com. The confidential randomization scheme was maintained in a computer isolated from the clinical team. Upon patient enrollment, the clinical team notified the study administrator who secured the sequence-appropriate assignment. This study was open (there was no blinding for patients, providers, or study investigators) and the randomization sequence remained concealed until study completion.

Trial inclusion/exclusion criteria were adopted from the earliest published study on acupuncture for CRF [18]. Inclusion criteria were that a patient is female, between 18 and 65, finished with primary therapy for breast cancer (free of cancer), and fatigued as measured by a score of 4 or greater on the Brief Fatigue Inventory (BFI) [18]. The exclusion criteria were depression (Hospital Anxiety and Depression Score [HADS] depression score >10), severe anemia at intake (Hemoglobin level <9 g/dL; hematocrit level <30; a decline in hemoglobin of 2 g/dL over the previous month; or in active treatment for anemia), or severe limitations in physical functioning (Kornofsky score <70). To maintain consistency with the inclusion/exclusion criteria from the earlier published study [18], we did not exclude patients with other potential causes of fatigue, such as hypothyroidism and hepatitis.

### Recruitment

We intended to meet a recruitment goal of 80 (based on a published power analysis [17]) by enlisting nearly 80 subjects from a prior study of symptoms conducted by a team member (SKS). However, we soon realized the need to diversify recruitment methods and tried several non-tailored recruitment methods. We ran newspaper ads, posted flyers in numerous clinics, cancer wellness centers, and support group meeting sites. We designed an oversized poster with formatted text/graphics and placed it in a heavily-trafficked hospital hallway. We posted an electronic announcement about the study in an email sent to approximately 700 institution-affiliated clinicians, researchers, and professors. Further, the study's Principal Investigator (KKH) sent a personal letter to 14 physician colleagues in which he requested referrals.

With the hope of developing a strategy to overcome continued difficulties with recruitment, we consulted the Director of the UCLA Survey Research Center. The Director recommended that we extend our recruitment efforts to an oncology waiting room (T. Hays, personal communication, July, 2008). We then worked on logistics with a clinic manager and nurse practitioner of an oncology practice that regularly refers patients to the clinic that was providing the study treatment (the UCLA Center for East-West Medicine). The clinic manager and nurse practitioner sought the support of their clinic's oncologists. These efforts resulted in a tailored recruitment strategy that involved enlisting oncologists into the research project and sending a recruiter to the oncology waiting room at predetermined times to enroll patients who were completing treatment.

### Intervention

A growing body of evidence suggests that interventions that are informed by theory, especially those combining multiple theories, are more effective than interventions that lack a theoretical basis [24]. A multidisciplinary team of medical researchers, acupuncturists, and physicians knowledgeable about patient education and acupuncture (MFJ, EKA, JJL, IK, KKH) developed our study's therapeutic protocol with reference to two bodies of theory: Social Cognitive Theory and Integrative Medicine. The team used Social Cognitive Theory to conceptualize and enhance self-efficacy, [36] which is defined as a person's confidence in her ability to perform a specific behavior [37]. Theorists consider self-efficacy to be the key catalyst of behavioral change [24]. Practitioners may use four distinct modes (ways) to help patients develop self-efficacy [36,38,39]. Additional file 1 draws upon Social Cognitive Theory literature [24,36-38,40] to communicate various ways in which IK, the study clinician who delivered patient education, sought to improve self-efficacy in patients. The "Assessment" column identifies mechanisms that IK used to assess patient adherence to self-care recommendations. The final column ("Mode of facilitating self-efficacy") provides a step-by-step accounting of the system the team developed to increase IK's fidelity to the self-care protocol across patients. In short, IK used the protocol to work with patients to develop individualized achievable goals accompanied by feasible tasks. This approach was designed to help patients more thoroughly implement recommendations; it has also been reported that this approach helps improve patients' confidence [39].

Our team used Integrative Medicine theory [26,27,41-43] to blend several non-pharmacologic options into a coherent protocol oriented towards addressing CRF in the context of healing the whole person. The team set, as a high priority, the development of a strong, positive clinician-patient bond by arranging for study acupuncturists to conduct in-depth, person-centered clinical intakes. During intake, CRF was treated as a signal pointing to clues for developing a wellness plan. Across all treatment sessions the study acupuncturists sought to help patients improve their self-care by tailoring advice according to TCM diagnosis, engaging in dialogue through presenting common ideas about well-being with a TCM-based perspective, and refocusing away from a quick fix mentality to a longer-term holistic perspective [26]. Conventionally trained oncologists, physicians and researchers provided substantial input as the team developed the treatment protocol.

The first four sessions of the therapeutic protocol involved IK administering self-care training alongside acupuncture. For the four weekly self-care sessions, IK sought to help patients enhance their general well-being through facilitating lifestyle changes in the areas of self-massage, exercise, nutrition, and stress management (relaxation)(Additional file 1). She also provided fatigue-specific recommendations in the form of dietary advice (Additional file 1). A second acupuncturist (JJL) provided the eight weekly acupuncture sessions. These were individualized according to one of four different symptom patterns of dysfunction that could trigger CRF and other problems. He also needled four energy-associated points (Table 1). The team anticipated that patients would require several weeks to incorporate effective self-care. Believing that acupuncture would produce more immediate benefits, the team provided education during the first four weeks of treatment rather than in the middle or at the end of the eight week therapeutic protocol.

**Table 1:** Extract from Acupuncture Protocol, presented in accordance with STandards for Reporting Interventions in Controlled Trials of Acupuncture (STRICTA)^68^

Item	Domain	Item description	Study-specific details
1	Acupuncture rationale	Rationale for selection of points and literature sources	The acupuncturist needled four energy-associated acupuncture points on all patients.^69^

2	Acupuncture rationale	Individualization and literature sources	Based on clinical experience and TCM theory,^69 ^the study acupuncturist [name] developed a system of classifying patients into one of four symptom pattern subgroups that may contribute to the development of fatigue symptoms: (A) gastrointestinal symptoms and signs, (B) emotional symptoms and signs, (C) sleep dysfunction symptoms and signs, and (D) myofascial muscle pain. Classification took account of patient's physiological dysfunctions as recognized by current state of health, previous disease conditions, and treatment history.

3	Needling details	Points used	For each patient, the study acupuncturist administered acupuncture at 4 energy-associated points (Li4, Sp6, ST36, Ki3) and then additionally needled acupuncture points he considered beneficial primarily in light of symptom pattern classification. Those in the gastrointestinal symptom pattern also received acupuncture on P6 and Sp4. Those in the emotional symptom pattern also received acupuncture on Lu7, Ki4, Liver3, Yintang, and Gv20. Those in the sleep symptom pattern also received acupuncture on H7, Ki4, and Ub62. Those in the pain symptom pattern also received acupuncture on Gb20, Te5, Gb43, SI3, Ub62, Gb29, Gb30, Gb40, and selected points related to specific sites of somatic pain. All points were administered bilaterally.

4	Needling details	Depths of insertion	Points were needled perpendicularly to a depth of 0.5-1.5, with exact depth varying by location of acupuncture point, ease with which patient achieved the "de qi" ("twitch") sensation, patients' size, sensitivity, and their state of health.

5	Needling details	Responses elicited	The study acupuncturist sought to achieve the "de qi" (twitch) response by employing needling techniques such as lifting, thrusting, and rotating.

6	Needling details	Needle retention time	Needles were inserted over the course of approximately 10 minutes, retained in place for about 30 minutes, and removed over the course of about 10 minutes (50 minutes total)

7	Needling details	Type of needles	LEKON^™ ^sterile disposable acupuncture needles of the following sizes: 34G (gauge) × 1.5 (0.22 mm × 40 mm diameter); 34G× 1.0 (0.22 mm × 25 mm); 32G× 1.5 (0.25 mm × 40 mm); and 38G× 0.5 (0.18 mm × 13 mm); needle types were chosen according to acupuncture point location and patients' size, sensitivity, and state of health.

8	Practitioner background	Duration of relevant training	The clinician delivering acupuncture [Jie-Jia Li] earned a medical degree from the Southern Medical University in Guangzhou, China, a Master's degree in Traditional Chinese Medicine from Shanghai University of Traditional Chinese Medicine, and a Ph.D. in Traditional Chinese Medicine at American Liberty University in California.

9	Practitioner background	Length of clinical experience	JJL has over 20 years of experience in providing acupuncture and has worked at the UCLA Center for East-West Medicine for over 15 years.

10	Practitioner background	Expertise in specific condition	JJL has been treating patients with breast and other types of cancer for nearly 10 years; he has been collaborating with [Name] in researching breast cancer for more than five years.

In brief, a multidisciplinary team utilized Social Cognitive Theory and Integrative Medicine Theory to develop a therapeutic protocol that would address fatigue in the context of caring for the whole person. To do so, the practitioners oriented patients towards a holistic view of health, built up their self-efficacy through a structured set of progressively challenging mastery experiences, and delivered acupuncture early on to achieve results that would enhance commitment to and confidence in self-care.

### Usual Care

Participants in both arms of the study received usual care from their personal physicians according to the principles of normal practice which included pharmacologic and non-pharmacologic options [2].

### Outcome

We pre-specified the study's primary outcome to be CRF as measured by the BFI at follow-up. The BFI is a scale composed of 9 items that assess intensity of present, usual, and worst fatigue during past 24 hours and interference with usual activities [18]. Response choices range from 0 (no fatigue) to 10 (worst imaginable fatigue) [18]. A single score ranging from 0 to 10 was computed for the BFI by averaging responses to all 9 items. Previous research provided evidence in support of the reliability and validity of the BFI [18]. Cut-off scores distinguish mild (1-3), moderate (4-6), and severe (7-10) CRF that correspond to the NCCN-recommendations for the CRF clinical practice guidelines screening question: "How would you rate your fatigue on a scale of 0-10 over the past 7 days?" [2].

To assess changes in cognitive complaints between first and last visit, we utilized the FACT-COG. This 50 item measure has 3 summary scores: cognitive problems, impact on functioning, and impact on health-related quality of life [44].

### Statistical Methods

We used an analysis of covariance (ANCOVA) model. We fit ANCOVA with Huber's M estimation, a robust regression methodology [45]. The regression equation models BFI score at follow-up as a function of an intercept and two variables: BFI score at baseline and treatment status (0 if patient randomized to control and 1 if to treatment). Following published recommendations on statistical analysis for Phase II trials, [46,47] we used a statistical significance criterion of p <0.10 for a two-sided test on the primary outcome variable. One participant was randomized to usual care treatment but received patient education and acupuncture. She experienced a favorable response. Adhering to the randomization protocol, we kept her in the usual care group.

### Human Subjects

Both the general and cancer-specific Institutional Review Boards at UCLA approved this study (#06-010-01, #POP0504401). All participants provided written informed consent.

## Results

### Patient Recruitment

**Figure 1:**
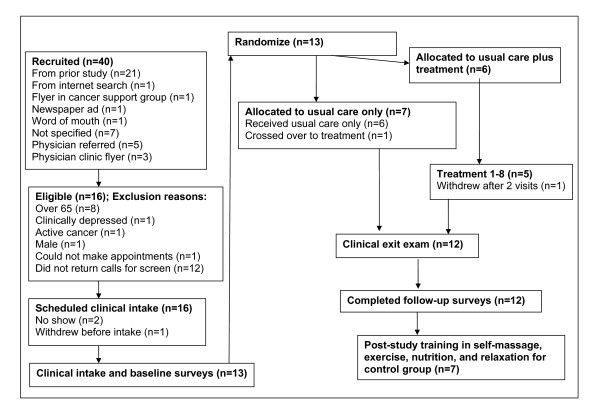
**CONSORT diagram**.

Over 15 months, 40 people contacted the recruitment manager concerning the study. Figure 1 details the flow of participants through the recruitment, randomization, and treatment processes. Thirteen candidates were eligible; seven were randomized to control and six to treatment. One person in treatment withdrew after two weeks (no reason given). We were unable to collect any further information from this subject.

We began recruiting subjects by mailing the 80 women who had participated in an earlier open-ended interview study a letter inviting participation. Over approximately three months, 21 indicated a willingness to join. Of these, 8 were eligible and 5 participated in the research trial.

Responses to the initial (non-tailored) recruitment methods were limited. One woman responded to the newspaper advertisements. Three women contacted the recruitment coordinator after reading posted flyers. We had no indication that either the oversized poster or departmental email resulted in participants. The principal investigator's personalized letters resulted in 5 subjects contacting the recruitment manager. In sum, the non-tailored recruitment these 5 methods led approximately 9 people to express interest in the study over the course of 12 months.

In contrast, the tailored recruitment strategy was much more fruitful. Over the course of two weeks, a recruiter conducted two two-hour recruitment sessions that resulted in five patients expressing interest in our study. Extrapolated to a 12 month period, the tailored recruitment strategy may identify more than 100 breast cancer survivors who are interested in participating.

### Outcome Results

Baseline demographic and clinical characteristics of the 12 study participants are shown in Table 2. There were no statistically significant differences between the treatment (n = 5) and control (n = 7) subjects in terms of sociodemographic and clinical indicators (independent group t-tests). This indicates that the randomization procedure did not fail to provide for a fair comparison between treatment and control. Overall, the mean age of study participants was about 54 and about two-thirds of the participants were white.

**Table 2:** Participant baseline characteristics

	Number of Patients		
Characteristic	Treatment group (n = 5)	Control group (n = 7)	p-value (two-tailed)
			
BFI	06.33 (1.39)	06.00 (1.09)	0.65
			
Age, years	55.00 (6.40)	53.00 (7.20)	0.58
			
Race			0.58
White	4	4	
Black or African American	1	3	
			
Income			0.47
< $20,000	0	2	
$20,000 or higher	5	5	
			
Marital status			~0.99
Married or cohabiting	3	4	
Single/divorced/separated	2	3	
			
Employment status			0.58
Employed	4	4	
Unemployed	1	3	
			
Chemotherapy			0.58
Yes	4	4	
No	1	3	
			
Radiation therapy			0.24
Yes	4	2	
No	1	5	
			
Hormone therapy			0.22
Yes	3	1	
No	2	6	
			
Severe pain			0.29
Yes	1	4	
No	4	3	

Note: p-values are from independent group t-tests.

The mean (standard deviation) scores of CRF, for the pre-specified primary outcome, CRF as measured by the BFI, were 6.33 (1.39) and 6.00 (1.09) at baseline for the treatment and control groups, respectively (See Table 3). This is close to a score of 7 which is considered "severe" fatigue. After the ten-week treatment period, the respective scores were 2.13 (1.23) and 4.38 (2.53). These correspond to "mild" and "moderate" fatigue in the treatment and control groups, respectively. The regression model estimates indicate that the treatment group patients benefited from a 2.38-point greater decline in CRF compared to the control group (See Table 4). The standard error of the treatment variable was 1.35 and the p-value 0.08. Thus, the study provides evidence in favor of a treatment benefit.

**Table 3:** Descriptive statistics

Variable	Treatment (n = 5)		Control (n = 7)	
	
	Baseline mean (SD)	Follow-up mean (SD)	Baseline mean (SD)	Follow-up mean (SD)
*Pre-specified primary outcome*				

BFI	06.33 (1.39)	02.13 (1.23)	06.00 (1.09)	04.38 (2.53)
*Secondary outcomes *(FACT-COG)				

Perceived cog impairments, QOL impact	02.70 (1.24)	03.55 (0.21)	01.58 (1.27)	02.21 (1.22)
Perceived cognitive impairments	03.00 (0.36)	03.34 (0.06)	02.02 (0.83)	02.27 (0.95)
Perceived cognitive abilities	03.23 (0.54)	03.51 (0.49)	01.86 (0.71)	02.33 (0.84)

**Table 4:** ANCOVA estimation of pre-specified primary, post-specified secondary, and ancillary outcomes

Outcome Variables	Treatment effect estimate	Standard error	P-value
*Pre-specified primary outcome*			

Brief Fatigue Inventory (BFI)	-02.38	01.35	0.0776
			
*Secondary outcomes*			

Perceived cognitive impairments, QOL impact(FACT-COG)	-00.44	00.56	0.4286
Perceived cognitive impairments (FACT-COG)	-00.16	00.30	0.6024
Perceived cognitive abilities (FACT-COG)	-00.02	00.45	0.9609

ANCOVA is fit with Huber's M estimation, a robust regression methodology.^44 ^Rregression equations model score at follow-up as a function of an intercept, pre-treatment score and treatment (an indicator variable).

A reduction in our pre-specified primary endpoint CRF as measured by the BFI is suggestive of a clinically meaningful outcome. Based on the baseline pooled standard deviation and post-treatment means, the effect size was 1.85. According to the Cohen's D metric, a large effect size is equal to or greater than 0.80 and a clinically meaningful difference is considered to be 0.20 or greater [48].

The evidence failed to suggest that the treatment decreased cognitive complaints.

The clinician performing acupuncture (JJL) was provided with a standardized checklist to assess for adverse events after each treatment, including bruising at needling site, panic, severe disorientation, fainting, infection, or puncture of an internal organ. The completed reports indicated that the acupuncture treatments were not associated with any adverse events.

## Discussion

Responding to requests from the oncology community for a multimodal and broad-acting therapy to address CRF, [7,8] we drew upon social cognitive theory and integrative medicine theory to assist patients with improving self-care, especially in terms of exercise and nutrition, in the context of providing acupuncture. The hypothesis that integrating patient self-care into treatment routines enhances clinical outcomes rests upon mixed findings [49]. One possible explanation for our positive results resides in the growing body of evidence suggesting that interventions informed by theory, especially those combining multiple theories, are more effective than interventions lacking a theoretical basis [24].

Participants in the treatment group experienced a 66% reduction in fatigue (see Table 4: (6.33-2.13) / 6.33). This result compares very favorably with similar studies examining acupuncture or acupressure in the relief of CRF [18-22]. We believe that our treatment protocol integrating patient education with acupuncture provides more benefit than acupuncture alone because our protocol enhances self-care and effective self-care produces substantial positive health benefits.

### Limitations

The foremost limitation of this study is the small sample size. We began the study anticipating that we would be able to recruit nearly 80 subjects from a previous study of symptoms in 80 breast cancer survivors. While succeeding in recruiting only 5 participants, we realized that such a strategy would not be dependable for future studies. Recognizing the need to diversify our recruitment methods, we tried several additional non-tailored approaches. In this process, we realized there are at least two distinct types of breast cancer survivors, each of which is reachable in very different ways. The first is patients finishing primary treatment and transitioning from the care of an oncologist to a primary care physician. The second is patients who have completely finished treatment and are seen solely by primary care physicians. The first could be identified at oncology clinics as they finish treatment while the second is a more geographically dispersed population requiring contact at a variety of recruitment sites including primary care clinics, cancer survivor group meetings, cancer survivor events, etc. We see recruitment of patients finishing treatment as much more feasible. Accordingly, we recommend that researchers interested in replicating our protocol focus on addressing CRF at the completion of active cancer treatment and invite oncologists to play a key role in study design, implementation, and result dissemination. In any case, thoughtful and proactive recruitment methods are needed for successful conduct of a larger trial.

There were several other limitations to our study. Our exclusion criteria were consistent with that of one study [18], but not others [e.g. [22]]. The self-care components of the protocol could have been more effectively monitored with standardized questionnaires that assessed changes in patient behaviors and responses to these changes. We did not examine any biological mechanisms through which the intervention might decrease CRF. Finally, the open (non-blinded) study design may have increased intervention efficacy

### Conclusion

Our study has provided evidence to support the feasibility of conducting a larger-scale RCT on patient education integrated with acupuncture to reduce CRF.

## Competing interests

The authors declare that they have no competing interests.

## Authors' contributions

MJ helped conceive the study, helped perform the statistical analysis, and drafted the manuscript. RDH helped conceive the study, helped perform the statistical analysis, and edited the manuscript. SKS helped conceive the study, helped recruit participants, drafted the section on recruitment, and edited other parts of the manuscript. RME directed the statistical analyses and edited the manuscript. EKA helped conceive the study, performed the clinical intakes and physical examinations, and edited the manuscript. JJL treated patients, participated in drafting the results section, and reviewed the manuscript. IK treated patients, participated in drafting the results section, and reviewed the manuscript. RBV participated in coordinating the study and edited the manuscript. JL conducted statistical analyses, drafted part of the results section, and reviewed the manuscript. LGY carried out data entry, organized the bibliography, and reviewed the manuscript. As Principal Investigator, KKH directed and orchestrated the project, and this manuscript, from conception to conclusion. All authors read and approved the final manuscript.

## Pre-publication history

The pre-publication history for this paper can be accessed here:

http://www.biomedcentral.com/1472-6882/11/49/prepub

## Supplementary Material

Additional file 1 **CONSORT Diagram**. A word document with a CONSORT diagram. Click here for file
