# Supplementary material for: Patient education integrated with acupuncture for relief of cancer-related fatigue randomized controlled feasibility study
Source: BMC Complement Altern Med. 2011 Jun 25;11:49. doi: 10.1186/1472-6882-11-49 (PMC3144009; doi:10.1186/1472-6882-11-49)
Supplement: Additional file 1 — CONSORT Diagram. A word document with a CONSORT diagram. [file 1472-6882-11-49-S1.DOC]

**Additional file 1.** Extract from Patient Self-Care Protocol

| **Item**  **Number** | **Encounter** | **Objective** | **Activities** | **Assessment** | **Mode of facilitating self-efficacy** (specific behavioral strategy) **[Arranged chronologically by time of delivery]** |
| --- | --- | --- | --- | --- | --- |
| 1 | Clinical Intake | Establish positive clinician-patient relationship | Western history; TCM pulse and tongue diagnosis; specific inquiries on fatigue | Clinician’s assessment at end of clinical intake | **Verbal persuasion** (CRF described as a signal pointing to clues for developing a wellness plan that clinician and patient can cooperate to develop) |
| 2 | Participatory Session 1 | Patient orally contracts to self-massage 5 specified acupressure points for a total of 20 minutes a day | Acupuncturist helps patient locate 5 points related to overall well-being and then teaches a simple stimulation technique (gently & slowly pressing up & down) | At Participatory Session 2, clinician dialogues with participant about and observes her self-acupressure | **Verbal persuasion** (acupuncturist describe the benefits of self-massage for health promotion and well-being); **Vicarious experience** (acupuncturist models self-massage on patient); **Performance accomplishment** (patient practices self-massage); **Verbal persuasion** (acupuncturist provides realistic positive feedback to patient about their self-massage); **Physiologic feedback** (patient learns to use physiologic feedback to distinguish when self-massage is helpful or not); **Performance accomplishment** (patient orally contracts to self-massage 5 specified acupressure points for 20 minutes a day) |
| 3 | Participatory Session 2A | Patient implements more advanced techniques into self-acupressure routine | Acupuncturist helps patient learn more advanced techniques (sliding press and rotating press) | At Participatory Session 3, clinician dialogues with patient about her self-acupressure and observes her self-acupressure | Same as Item 2 (above); also: **Verbal persuasion** (acupuncturist provides verbal encouragement to reinforce self-change); **Physiologic feedback** (acupuncturist helps participant appropriately associate self-acupressure to changes in physiological states) |
| 4 | Participatory Session 2B | Patient cooperates with acupuncturist to develop a realistic individualized goal for exercise | Acupuncturist describes several different non-strenuous exercise routines, including potential benefits and problems; this discussion is tailored to account for patient’s individual health condition | At Participatory Session 3, patient reports to clinician about exercise | **Performance accomplishment** (develop a goal for exercise); **Vicarious experience** (Participants were given the option to view video clips with acupuncturist of specific exercise routines, such as Tai Chi); **Vicarious experience** (acupuncturist may model specific exercise components); **Verbal persuasion** (acupuncturist describes specific benefits of exercise routines for fatigue and well-being); **Physiological feedback** (acupuncturist identifies which physical discomforts are signs that exercise is too strenuous) |
| 5 | Participatory Session 3 | Patient cooperates with acupuncturist to develop realistic individualized goals for improving intake of food that is nutritious, healthily prepared, and mindfully consumed | Acupuncturist describes nutritious food selection, healthy food preparation techniques and mindful food consumption manners | At Participatory Session 4, patient reports to acupuncturist about food | **Performance accomplishment** (develop a goal and prescription for healthy eating); **Vicarious experience** (Acupuncturist carries out a role play on mindful eating); **Verbal persuasion** (acupuncturist describes the positive impact on relief of fatigue for specific food selections: eating oatmeal for breakfast, incorporating barley into the diet, and eating healthy servings of broccoli, sweet potatoes, and cabbage); **Physiological feedback** (acupuncturist identifies the ways in which routines such as eating at regular times and atmosphere such as no television will help to relieve fatigue) |
| 6 | Participatory Session 4A | Patient cooperates with acupuncturist to identify a relaxation technique to incorporate into her lifestyle | Acupuncturist teaches simple relaxation techniques | Patient self-assessment | **Performance accomplishment** (develop a goal and prescription for relaxation); **Vicarious experience** (Acupuncturist demonstrates relaxation technique(s) of interest; **Verbal persuasion** (acupuncturist describes how relaxation may reduce fatigue); **Physiological feedback** (acupuncturist helps participant to develop a mindful awareness of the ways in which practicing relaxation will help to relieve fatigue) |
| 7 | Participatory Session 4B | Positive closure | Acupuncturist reviews overall protocol on integrative medicine lifestyle changes and reinforces potential benefits | N/A | N/A |
